# Maternal Nutrition Education Provided by Midwives: A Qualitative Study in an Antenatal Clinic, Uganda

**DOI:** 10.1155/2018/3987396

**Published:** 2018-10-25

**Authors:** Joyce Nankumbi, Tom Dennis Ngabirano, Gorrette Nalwadda

**Affiliations:** Department of Nursing, College of Health Science, Makerere University, Kampala, Uganda

## Abstract

Maternal nutrition during pregnancy affects the health of the mother and baby. The objective of this paper is to describe the maternal nutrition education offered by midwives to women attending an antenatal clinic. The study also examined the resources, support, and the needs of the midwives in offering the nutrition education. Six in-depth interviews with the midwives, six direct structured observations of the group education, and 12 one-on-one interactions of midwife and pregnant women observations were completed. The interviews and field observation notes were typed and analyzed using the latent content analysis. The emerging themes were the maternal nutrition education and the education needs of the midwives. The content and presentation of maternal nutrition were inadequate in scope and depth. The maternal nutrition education was offered to only pregnant women attending the first antenatal care visit. The routine antenatal education session lasted 45 minutes to 1 hour, covering a variety of topics, but the nutritional component was allotted minimal time (5–15 minutes). The organization, mode of delivery, guidelines, resources, and service environment were extremely deficient. The relevance of appropriate weight gain during pregnancy, guidelines for healthy habits, avoidance of substance abuse, and nutrition precautions in special circumstances was missing in the nutrition presentation. Information, maternal nutrition education resources, infrastructure, and health system gaps were identified. There was an inefficient nutrition education offered to the pregnant women attending the antenatal clinic. As means of promoting effective nutrition education, appropriate in-service training, mentorship, and support for the midwives are needed, as well as infrastructural and resource provision.

## 1. Introduction

Pregnancy is a time of intensified nutritional vulnerability, and the nutritional status of women before and during pregnancy can have a substantial influence on fetal and maternal outcomes [1]. The importance of maternal nutrition to fetal development and birth outcomes has been investigated [2–4]. Maternal nutritional deficiencies have been related to adverse maternal and birth outcomes including anemia, premature delivery, low birth weight, and other morbidities [5, 6].

Annually, 800,000 neonatal deaths though small for gestation age at birth, stunting, and wasting are linked to maternal undernutrition [7, 8]. The situation is even worse in low-resource countries like Uganda where maternal malnutrition increases the disease burden, having a major impact on the health of the mother and the child. In Uganda, malnutrition among women and children remains a public concern regardless of food availability. Uganda is among the top 20 countries worldwide with a high burden of undernutrition [9]. As means of intervention, studies have reported that nutrition education was found to have a positive effect on the nutrition awareness of the pregnant women [10, 11] which significantly contributes to improved nutrient intake (Ota et al., 2015). In fact, other studies have also established that nutrition education has significant association with good pregnancy outcomes [12–14]. Antenatal care (ANC) is a key entry point for pregnant women to receive a broad range of health promotion and preventive health services, including nutrition education and support [15, 16]. However, the nutrition education offered during the antenatal visits has been found to be insufficient [15]. This could be due to inadequate training of the midwives and other health professionals [17, 18].

In low- and middle-income countries, including Uganda, nutrition education is seen to be an appropriate intervention for health improvement [19–21]. Further still, the Uganda maternal and child nutrition guidelines 2010 recommend that the pregnant women should be educated on the importance of adequate nutrition, relevancy of weight gain, increased nutrient requirements, nutrient-rich dietary sources, importance of micronutrient supplementation, appropriate food preparation, and safety and hygiene methods [22]. However, there is a lack of a documented evaluation of the maternal nutrition education offered during the antenatal care, and whether the national nutritional guidelines have been implemented as stipulated. Therefore, this paper documents the characteristics of the maternal nutrition education offered to pregnant women during the antenatal care (ANC) visits and the challenges encountered by the midwives.

## 2. Methods

### 2.1. Study Design

A qualitative description approach was used based on the fact that there were high levels of uncertainty about the current education intervention and very little existing research on prenatal nutrition education in Uganda and at the same time limited resources to conduct the study [23]. The authors conducted six in-depth interviews with midwives who were involved in the direct care of the pregnant women at the ANC clinic. In-depth interviews are useful and appropriate for obtaining detailed information about a person's thoughts and behaviors and often provide context to other data [24, 25]. In addition to the in-depth interviews, nonparticipant-structured observations of the nutrition education sessions during the routine ANC visits were conducted. The study included sessions conducted by the midwives, and this was the routine at the clinic. The observations included six group education sessions and twelve one-on-one interactions between the midwives and the pregnant women during the physical examination in the antenatal clinic.

### 2.2. Study Setting

The study was conducted at an ANC clinic at a referral and teaching hospital in Uganda, and it is one of the busiest maternity units in the country. The ANC is offered to women of various ethnicities, socioeconomic classes, and religions. The clinic operated from Monday to Friday from 8:00 am to 5:00 pm. It offered antenatal services including HIV counseling and testing, syphilis screening, health education that included nutrition education, screening, and examination, and referral services. The clinic was structured in two sections: a low-risk unit which was attended by women with good obstetrical history and a high-risk unit which was attended by women with poor obstetrical history. The clinic is predominantly staffed by midwives. At the time of the study, a total of 2,155 mothers had attended the antenatal clinic between January and March 2015, and the clinic had 29 midwives.

### 2.3. Study Procedure

In this study, the researchers looked out for any set of planned educational activities that involved teaching pregnant women about nutrition, providing educational materials that reinforced messages about healthy eating, taught nutritional skills essential for making a dietary change, and provided information on how to sustain the changed behavior. Group nutrition education included sessions where the midwife gave nutrition education to a group of pregnant women attending ANC. One-on-one interactions included an interface between a midwife and a pregnant woman with the intention of antenatal nutritional education [26].

The group education session observations were conducted in the morning hours of the antenatal clinic and the one-on-one interactions between women and the midwives continued throughout the day. The observations recorded information on the hospital education environment, organization of the sessions, conduct of the sessions, content of the nutrition class, evidence of planning and preparations for the education activities, documentation done by the midwife of the nutrition education activities, and the resources and the information education and communication (IEC) materials available at the clinic. The observations also included verbal behavior, body language, and objects or resources used during the nutrition education sessions. Midwives were observed during the formal teaching, during the informal teaching, and during the examination of the pregnant women. The researchers observed 6 group education sessions, and each of the midwives was observed at least twice during the informal education or examination sessions. The midwives conducted the education sessions in the local language, and however, the notes taken by the researcher were in English, and the researcher was well conversant with the local language and was able to take notes in English. The in-depth interviews were conducted in English.

In-depth interviews and observation were conducted in January and February 2016. The researchers introduced themselves to the area managers and the in-charge nurse of the hospital antenatal care clinic. The purpose and procedure of the study were explained to the midwives in order to obtain permission for conducting the study in the clinic. After informed written consent was obtained, the researcher together with the midwife identified a time that was convenient for the in-depth interviews. An open-ended in-depth interview guide was used for data collection. This majorly focused on how the sessions are given, and the midwives' challenge in giving the nutrition education. An appropriate time and setting for the interview were identified by the participant. The in-depth interview lasted between 30 and 60 minutes, the interviews were tape recorded, and the notes were also taken. A structured nonparticipant observer method allowed the researcher to look for specific behavior without interfering in the education sessions. The observer was visible and known to the study participants.

### 2.4. Ethical Considerations

Ethical review and approval were obtained from the Makerere School of Health Sciences research ethics committee (REF: 2015–026). The hospital gave the researchers administrative clearance to conduct the study. Informed written consent was obtained from the study participants. Confidentiality and privacy were maintained throughout the study period. The data collected were strictly for the purpose of this research, which were anonymized in the writing of this article. The audio data will be stored for 5 years after which they will be destroyed. The data files are stored in a computer with a password to allow only access by the researchers.

### 2.5. Data Analysis

Data from the in-depth interviews were transcribed verbatim. Notes were also taken during the structured observations. Microsoft word files for the interviews and observations notes were created. The files were password protected and were saved in a portable computer only accessible to the researchers. After the Microsoft word files were ready, all the researchers got copies and analysis began at an individual level. After an understanding of the data, the researchers met several times and a group data analysis was also done. The data were analyzed manually using latent content analysis (Hsieh and Shannon, 2005). The researchers used an interview or an observation as the unit of analysis for coding. This means that the data were not coded sentence-by-sentence or paragraph-by-paragraph but coded for meaning [27]. The transcripts were read and re-read by the researchers to develop coding categories, and a code book was developed to ensure consistent application of the final coding categories. The data were then coded independently by the researchers. The codes were later reviewed by the researchers to ensure agreement and consistency of meaning in situations where differences arose. The agreed upon codes were synthesized and grouped into exhaustive subcategories which were then merged into categories/themes. These represented the most common issues that emerged in the interviews and observations [5].

## 3. Results

There were two themes that emerged from the data. These are nutrition education in a hospital setting and education needs of the midwives.

*Theme 1*. Maternal nutrition education offered to pregnant women during the antenatal care visits.

Three subcategories under this theme were identified, and they included clinic environment and organization of the education sessions, mode of delivery of the education sessions, and resources available for the education (Table 1).

### 3.1. Clinic Organization and Environment for Maternal Nutrition Education Sessions

The maternal nutrition education sessions were integrated with the routine daily health education and promotion activities. The antenatal education was offered to pregnant women who are attending ANC for the first time in the current pregnancy. The pregnant women who were attending ANC for the 2nd to 4th visit were asked to proceed to different service points excluding the education point. The targeted pregnant women were identified by the midwife and moved to the venue/education point. The venue had both permanent seats and portable benches and was occupied by the women all the times. The venue was not spacious; however, it had adequate ventilation. Pregnant women who attended with their spouses were given a special corner for more comfort during the education. Only about 1% of the participants were men. Sixty to 80 individuals attended the daily session.

The routine topics of the education sessions that were picked out form the observations included maternal nutrition, the importance of antenatal care attendance, HIV counseling and testing, and danger signs in pregnancy. Two to five topics were covered in each session that lasted between 45 and 60 minutes. The time spent on the maternal nutrition education component ranged from 5 to 15 minutes. This was also confirmed by the in-depth interviews with the midwives.

“We conduct the education talk in group sessions – we just take very few minutes maybe about 15 minutes [on nutrition]. We try to conduct the talks daily – because we have sessions in the morning we just combine the whole picture but not emphasize on nutrition only.” (Interview with a diploma midwife)

Quite often, the sessions were interrupted by pregnant women joining late, as well as other persons passing by to different service points within the clinic. The seats were limited, and for some cases, pregnant women were observed attending the education sessions while standing, and sometimes pregnant women would walk away due to a lack of space to accommodate all the pregnant women. In such situations, it was observed that the midwife rushed the session to reduce the time that pregnant women spent standing. All the pregnant women were educated in a single group irrespective of their sociodemographic characteristics such as parity and age.

The education sessions were conducted by one or two midwives in the local language (Luganda). At the beginning of the sessions, the midwives introduced themselves to the women and then took on the authoritative teacher position. For all the observed sessions, the midwives did not have any form of visible preparation, lesson plans, or documentation of the education sessions. The clinic did not have any teaching aids, videos, visual aids, or any teaching materials to enhance the learning or understanding of the concepts by the pregnant women.

### 3.2. Mode of Deliveries of the Nutrition Education Sessions

The midwives educated women using a direct verbal instruction method. During the observation period, no practical demonstrations for the maternal nutrition education session were observed. During the in-depth interviews, the midwives reported that while it would be more practical and useful, they could not perform any demonstrations due to lack of resources. One-on-one maternal nutrition education sessions were reported to be conducted alongside the ANC examination when deemed necessary by the midwife, depending on the woman's nutrition status, for example, when the woman appeared malnourished or reported a poor appetite.

“Commonly we do a group session, but then we normally do one-on-one when there is need— maybe like you have spotted out a mother who really is anemic– so you need to take your time with her and you talk one on one with her.” (Interview with an enrolled midwife).

“*Actually we are supposed to be giving the oral presentation with the demonstration. But when I look at the antenatal clinic here; we do not have the resources for the demonstrations. So we just give the information; tell them the various food groups… … Otherwise, it would have been actually more practical and very helpful if we have the resources for demonstration.” (Interview with a diploma midwife).*

There was no observable evidence of content documentation of the education sessions at the clinic, and specifically, there was none for maternal nutrition. The teaching was dominated by the midwife, providing information rather than active participation by the pregnant women.

During the one-on-one interaction, there was direct communication between a woman and the midwife and the communication at times included maternal nutrition education. Midwives were noted to spend 5 to 10 minutes with the women including clinical examination of the woman. During the interaction, nutrition supplementation such as iron and folic acid was often mentioned and prescribed. Privacy during the examination and one-on-one interaction were audio and visually compromised.

### 3.3. Resources for Maternal Nutrition Education during Antenatal Care Visits

The setting lacked education resources such as visual aids, television screen, videos, flyers, or written IEC materials. This was also emphasized by the midwives during the in-depth interviews. Midwives were not aware of the existing maternal nutrition guidelines that could be used in packaging and the delivery of the maternal nutrition education messages to pregnant mothers.

“I am not aware of any guidelines for maternal nutrition… … since I came here in 2010; at least I have not seen them.” (Interview; midwife with 20 years' working experience).

*Theme 2*. Nutrition education needs of the midwives.

Under this theme two subcategories emerged: information and resource needs (Table 2).

### 3.4. Information Needs of the Midwives

Midwives utilized nutritional knowledge acquired during their preservice trainings, as long as 20 years before, which they felt was inadequate for sufficient current nutrition care of the women. Only a few midwives had received in-service training on nutrition but not specific to prenatal nutrition. Most midwives could not recall having had any specific training in maternal nutrition as depicted in the narrative below.

*The time I have been in this area… . we are using the knowledge which we had at the beginning and maybe adding some continuing medical education (CME) we have been attending, but not directly on maternal nutrition. (Interview with an enrolled midwife*).

The midwives also identified topics/areas of interest for additional training and skills to improve the nutritional care of the pregnant women. Among these, they included maternal nutrition during pregnancy; diet during and after pregnancy; food preparation methods that conserved nutrients; micronutrient supplementation such as iron, deworming, and nutritional disorders in pregnancy and their management; and managing a mother with other nutritional challenges. They also expressed a need for training on the current existing maternal and child nutrition guidelines.

“I would like to be educated on nutritious diets for pregnant women that can be prepared to preserve nutrients; I want to see how this ferrous we are giving – how it is working in their bodies” (Interview with a diploma midwife).

“I think I can benefit more if we would get someone to guide us through the existing guidelines such that we do not teach just as if singing a chorus of what we learned in the training school.” (Interview with a graduate midwife).

“I would like to receive training on diet during pregnancy – it seems good.” (Interview with an enrolled midwife).

### 3.5. Resource Needs for Maternal Nutrition Education

Resource needs reflected the problems of the current health system such as the need for additional time with the patient and increased staff to care for the extraordinary high clinic attendance of pregnant women. Little time is spent with the women in a response to handle the load. This has an implication on the human resource.

“We barely spend time with the women because you have to clear the line, and at the same time when you are teaching there are no resources for teaching … …” (An interview with the diploma midwife)”

Educational materials such as job aids, visuals, videos, television screens, written materials, and maternal nutrition guidelines were identified for improved maternal nutrition education. The midwives identified the need for more support from administrators and the Ministry of Health. The support included updates on current information and training where gaps had been identified for improved maternal nutrition care during pregnancy.

“I wish the ministry of health would orient us through the guidelines, and also have some of the basic resources such as charts, actual food models for demonstration during the teaching

## 4. Discussion

The paper describes the maternal nutrition education offered to pregnant women in a large antenatal clinic. It highlighted the maternal nutrition education needs for improved nutrition care and support for pregnant women. The antenatal period is the only time when midwives spend a significant time with the women in the reproductive age group and is an ideal time to provide nutrition education (Arish, Yeatman, and Williamson, 2013). In our setting, preconception nutrition is still a growing field. In this particular study, maternal nutrition education was found to be inadequate. The nutrition education sessions were characterized by very large groups of women, limited time for the teaching, educating women only once during their first antenatal care visit, and minimal participation by the women in the education session. There was a lack of documentation of the education sessions, lack of evidence of planning for the sessions, limited space for the teaching, and inconsistent messages by the midwives. In addition, women were educated in groups with no consideration of special characteristics such as age or parity. This increases the difficulty in identification of women with special nutrition needs and tailoring the message to such needs [4, 28]. In a setting like Uganda, with the inability to focus on women with special needs, improvement in nutrient intake may not be realized. It is a reality that there is a growing need to provide better training of the midwives and the health professionals at large in nutrition for better patient care and health promotion. The provision of inconsistent messages signified a need for training of the midwives about maternal nutrition. It is from such training that the midwives will appreciate strategies in providing nutrition education to women or education of large groups of participants. A paper by Kris-Etherton et al.(2014) also documents the dire need for the incorporation of the nutrition education competencies in the curricula of health professionals [29]. In low-resource settings like Uganda, nutrition education is seen as a health-promoting intervention and as the most feasible intervention to sensitize mothers about nutrition in pregnancy [11]. This implies that if the education is compromised, the consequences of poor nutrition will be realized. Furthermore, it is also always important to recognize that pregnant women attending ANC have different levels of nutrition knowledge. First, time pregnant women and adolescents may need more information, time, and attention than women who have had a pregnancy before [30, 31]. Only women attending the clinic for the first time were educated. This indicated a missed opportunity for follow-up and reinforcement of the nutritional knowledge. At the same time, the messages could be packaged for various sessions to be better understood and appreciated by the attending pregnant women. For the maternal nutrition education to be effective and adequate, the messages have to be right, consistent, and delivered with appropriate methods and strategies [32, 33]. Studies show that client education strategies that maximize good outcomes include the emphasis on what is necessary, choosing the right time, looking for teaching moments, and planning the education during uninterrupted time. In the same studies, it was noted that it is also important to evaluate the sessions, keep expenses in mind, clearly define goals and objectives, and document the nutrition education sessions and the outcome [34, 35]. It was noted from the findings of the study that there were scant resources for the successful conduct of the nutrition education sessions and support for MNE. This could be that the midwives are not competent to conduct the nutrition education sessions and in that case, resources are not lobbied for. Studies elsewhere have also reported inadequate resources to conduct nutrition education [28, 33, 36].

### 4.1. Limitations for the Study

Based on the study design, the results of this study may not be generalized to other settings. Reactivity by the study participants was also difficult to rule out given that the consent of the midwives was obtained before the observations. The topic of study had limited available literature; however, we did utilize the available information on the subject area. For future research, it would be relevant to put the basic resources in place and quality improvement studies to be done. In addition, a quantitative study could also be done to focus on the numbers.

## 5. Conclusion

The maternal nutrition education given to pregnant women during antenatal care was inadequate in scope and depth. The education was characterized by minimal time, inadequate space, interrupted teaching, and lack of documentation. In addition, the nutrition teaching was provided only once to the pregnant women at their first visit to ANC. The results of the study highlight gaps in the maternal nutrition education in a hospital clinic setting even with well-designed national nutrition guidelines. It is important that not only guidelines are formulated, but these guidelines are shared with the practitioners, in-service education is provided, and additional assistance is given in the implementation specifically including adequate education resources.

## Figures and Tables

**Table 1:** Maternal nutrition education in the hospital ANC.

Codes	Subcategory	Category/theme
Permanent seats in education venue	Clinic environment	Nutrition education in a hospital setting
Waiting area used for sessions		
Noise and interference during the group session		
A nonconducive learning environment		
Women continuously joining the sessions		
Midwife organized women to settle down for session		
Sessions conducted daily		
One-on-one teaching when necessary		
No preparation and documentation for the sessions		
Mandatory attendance at the first visit		
60–80 pregnant women and occasionally spouses attended the group session		
Starting time of the session was 8:00 am		
No audio aids	Availability of guidelines and nutrition education resources	
No use of visual aids in the session		
The absence of job aids, posters, and charts		
No videos/TV screen for ANC messages		
No fresh foods or demonstration of food preparation		
The absence of maternal nutrition guidelines for reference or guidance		
Group sessions	Teaching strategies	
The midwife stands in front of the mothers and give information, no demonstrations		
The one-on-one session only when necessary		
Minimal active participation by the mothers		
Limited time to ask question		

**Table 2:** Nutrition education needs of the midwives in the antenatal clinic.

Codes	Subcategory	Theme/category
How ferrous works	Information needs	Maternal nutrition education needs
Nutrition in pregnancy information		
Guidance through the MOH nutritional guidelines		
Information on diet during pregnancy		
Information on deworming and nutrition		
Information on nutrient conserving food preparation methods		
Information on nutritional disorders in pregnancy		
Lack of videos and television screen	Resource needs	
Actual foods for demonstration		
Inadequate time		
A large attendance of women at the clinic		
Lack of supply of relevant materials		

## Data Availability

The data used in the writing of this manuscript can be provided on request.

## Abbreviations

ANC: Antenatal care
MNE: Maternal nutrition education
IEC: Information, education, and communication materials.
